# Ecological Modeling from Time-Series Inference: Insight into Dynamics and Stability of Intestinal Microbiota

**DOI:** 10.1371/journal.pcbi.1003388

**Published:** 2013-12-12

**Authors:** Richard R. Stein, Vanni Bucci, Nora C. Toussaint, Charlie G. Buffie, Gunnar Rätsch, Eric G. Pamer, Chris Sander, João B. Xavier

**Affiliations:** 1 Computational Biology Program, Sloan-Kettering Institute, Memorial Sloan-Kettering Cancer Center, New York, New York, United States of America; 2 Immunology Program, Sloan-Kettering Institute, Memorial Sloan-Kettering Cancer Center, New York, New York, United States of America; University of Zurich and Swiss Institute of Bioinformatics, Switzerland

## Abstract

The intestinal microbiota is a microbial ecosystem of crucial importance to human health. Understanding how the microbiota confers resistance against enteric pathogens and how antibiotics disrupt that resistance is key to the prevention and cure of intestinal infections. We present a novel method to infer microbial community ecology directly from time-resolved metagenomics. This method extends generalized Lotka–Volterra dynamics to account for external perturbations. Data from recent experiments on antibiotic-mediated *Clostridium difficile* infection is analyzed to quantify microbial interactions, commensal-pathogen interactions, and the effect of the antibiotic on the community. Stability analysis reveals that the microbiota is intrinsically stable, explaining how antibiotic perturbations and *C. difficile* inoculation can produce catastrophic shifts that persist even after removal of the perturbations. Importantly, the analysis suggests a subnetwork of bacterial groups implicated in protection against *C. difficile*. Due to its generality, our method can be applied to any high-resolution ecological time-series data to infer community structure and response to external stimuli.

## Author Summary

Recent advances in DNA sequencing and metagenomics are opening a window into the human microbiome revealing novel associations between certain microbial consortia and disease. However, most of these studies are cross-sectional and lack a mechanistic understanding of this ecosystem's structure and its response to external perturbations, therefore not allowing accurate temporal predictions. In this article, we develop a method to analyze temporal community data accounting also for time-dependent external perturbations. In particular, this method combines the classical Lotka–Volterra model of population dynamics with regression techniques to obtain mechanistically descriptive coefficients which can be further used to construct predictive models of ecosystem dynamics. Using then data from a mouse experiment under antibiotic perturbations, we are able to predict and recover the microbiota temporal dynamics and study the concept of alternative stable states and antibiotic-induced transitions. As a result, our method reveals a group of commensal microbes that potentially protect against infection by the pathogen *Clostridium difficile* and proposes a possible mechanism how the antibiotic makes the host more susceptible to infection.

## Introduction

The intestinal microbiota has been receiving much attention lately. Recent studies, propelled by metagenomics and next-generation DNA sequencing technologies, establish novel connections between the intestinal microbial species composition and diseases [1]–[3]. An imbalance in bacterial composition has been linked to chronic diseases such as obesity [4], Crohn's disease [5] and type 2 diabetes [6]. Even drug-induced transient changes in the microbial community can increase the risk of developing diseases such as acute intestinal infections [7], or pulmonary viral infections [8] in mammalian hosts.

Although its importance has long been acknowledged [9]–[12] studies of the microbiota had been limited by the fact that most microbes are uncultivable in the lab. The recent developments in metagenomic high-throughput sequencing allow this by enabling the investigation of species composition directly without the need for culturing [13]. This has opened a new window into the microbial ecosystem residing in the intestinal tract. Our present view is that the intestinal microbiota is a relatively resilient ecosystem [14], with a composition that is quite stable over time [15], [16]. External perturbations, such as dramatic changes in diet [17] or antibiotic administration [18], can shift the composition. For example, broad-spectrum antibiotics can remove highly abundant species and allow less abundant, antibiotic-tolerant bacteria to dominate [7]. Antibiotic-induced losses of biodiversity increase the risk of bacterial infections [19], [20] and the effects can persist for several days after antibiotic treatment [18], [19], [21]. Perturbation-induced composition shifts are often observed in multispecies microbial ecosystems and may affect macroscopic overall functionality [22]. The loss of protective species can be resolved by reintroducing normally resident (or commensal) microbes. Faecal transplantation, i.e. the reestablishment of a patient's intestinal microbiota by introducing the microbiota of a healthy donor, is highly effective against *Clostridium difficile* induced colitis [23], [24]. Similarly, the reintroduction of anaerobic flora with high levels of *Barnesiella* sp. can clear intestines from highly abundant vancomycin-resistant *Enteroccocus* in mice [25].

**Figure 1:**
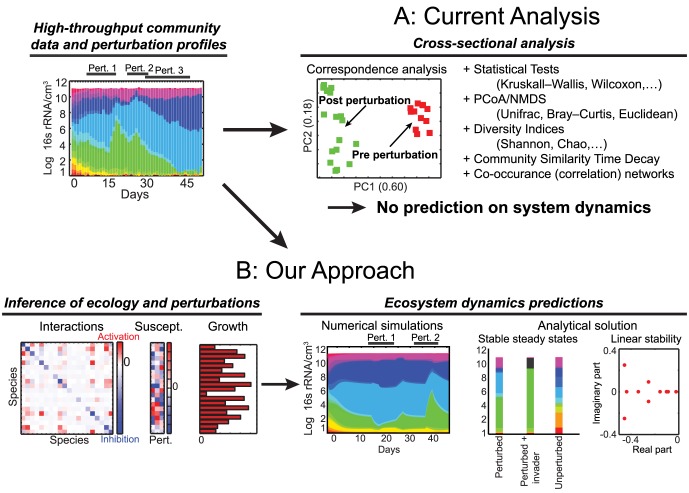
Conceptual figure highlighting the difference between our approach and the currently available methods for microbiota analysis. Used input data are the temporal records of microbial total abundances (colored bars on left) and the temporal signal of external perturbations (e.g. presence/absence or concentration). (A) Example and list of current computational approaches used to analyze community data for microbiota studies. (B) Our approach uses ecological modeling to infer a network of microbial interactions, susceptibilities to external perturbations and growth rates. The inferred parameters are used in an ecological community model which can then be used to predict ecosystem dynamics and to identify steady states.

In order to understand how commensal consortia confer resistance against pathogens it is crucial to identify the network of interactions between the species [26]. Interactions can be mediated by a direct secretion of substances such as bacteriocins [27], or ecological competition between the microbes [28], or even indirect interactions through immune system modulation [29]. Most quantitative studies of the intestinal microbiota so far focused on comparing the composition of different samples using quantitative indices and correspondence analyses [14] and cross-sectional statistical tests [1], [30]. Likewise, associations between microbial species are often obtained using correlation-based algorithms [26], [31]–[36], which results in undirected interaction networks. Singular value decomposition [28] or mixture model engines [37] allow for individuating stereotypical modes of response to external perturbations (i.e. grouping species positively or negatively affected by the stimulus) but they provide no information on the interactions themselves (Figure 1A).

We recently introduced an ecological model of microbiota dynamics that considers both species interaction networks and extrinsic perturbations such as antibiotics [28]. The model can explain how relatively simple ecological interactions such as competition for nutrients can lead to complex phenomena as, for example, multi-stability or antibiotic-mediated catastrophic shifts. Importantly, we concluded that quantitative knowledge of the microbial interactions could enable the prediction of microbiota dynamics. Predictive models can be of great therapeutic value by guiding antibiotic selection to reduce the risk of antibiotic-induced enteric disease [20]. However, no study to date has generated predictive models of ecological interactions and antibiotic perturbations.

Inspired by work on interaction inference in cheese-associated microbial communities [38] we extend the generalized Lotka–Volterra equations [39], [40] to infer microbiota ecology and predict its temporal dynamics under time-dependent external perturbations. A related approach based on linear ordinary differential equations has already been applied to gene-interaction networks [41]–[44]. Specifically, our method enables the quantification of (1) growth rates of microbial species, (2) species–species interactions, and (3) susceptibilities of microbial groups to time-variable external perturbations such as antibiotics. Moreover, we can use these parameters to numerically predict dynamics of the microbiota and to characterize its stability (Figure 1B). Using this method, we analyze data from a recent mouse study [19], which shows that the antibiotic clindamycin increases susceptibility to *Clostridium difficile* colonization. Our results suggest the existence of resilience and multistability in the intestinal microbiota and lead to a hypothesis on a subnetwork of microbial groups involved in the native resistance against pathogen colonization. This study demonstrates that data-derived models of microbiota dynamics can have significant analytic and predictive power. As such, inference and prediction algorithms could be used in combination with metagenomics to assist in the rational design of therapies such as antibiotic or probiotic therapies [12].

## Results

### Inference of ecological microbiota dynamics from time-series data

Extracting model parameters using a time-discrete Lotka–Volterra system has already been presented in the context microbial communities [38], [45], [46]. We extend this approach by introducing time-variable perturbations and applying Tikhonov regularization to solve the discretized Lotka–Volterra equations. Furthermore, we use the obtained parameters to predict dynamics and assess the system's stability.

In this spirit, we adopt the general deterministic approach of modeling time-dependent ecological dynamics using generalized Lotka–Volterra equations [39] with the addition of external perturbations. Formally, this model consists of autonomous, non-linear, coupled first-order ordinary differential equations,
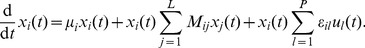
(1)Here 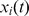 is the concentration of a focal species 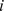, 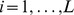, at time 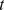, 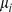 is its specific growth rate, 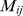 is the effect of the interaction of species 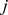 on species 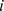 and 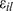 is the susceptibility to the time-dependent perturbation 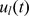 (for instance, an antibiotic or diet).

Ecological time-series data, such as longitudinal metagenomic sequencing data [15], [47], provide the composition of a community at discrete time points. Temporally resolved metadata, such as the timing of antibiotic administration [20] or of changes in diet regimes [17], may also be available and provide information about processes that perturb the microbiota. In order to translate the time-discrete data to a time-continuous dynamical system we divide (1) by 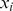 and discretize (see Materials and Methods),
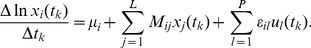
(2)The model parameters are determined by a linear system of equations, which is then solved using Tikhonov regularization [48] in order to ensure uniqueness and stability of the solution,
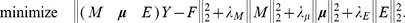
(3)The values for the regularization parameters 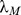, 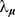, 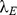 can for example be found in 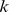-fold cross-validation (we use 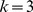) as the minimizer of the mean-squared stepwise prediction error to set the optimal trade-off between data fit and robustness towards the introduction of unseen or missing data [49].

The inference method was first tested on *in silico* data by generating trajectories for a Lotka–Volterra model as defined in (1). We created multiple trajectories of ecological systems characterized by different population sizes, random growth rates, interaction values and susceptibility parameters while ensuring system stability [50], 51. The simulations were also subjected to random perturbations of variable duration and white noise was added to simulate measurement uncertainty (Figure S1). The test confirms that the minimum of the stepwise prediction error can be used as a suitable proxy for the minimization of the parameter inference errors (Figure S2). Given the inferred parameters we can now predict the temporal dynamics by solving (1). We applied this approach to *in silico* data. The results are presented in Figure S3.

### Microbial interactions, antibiotic perturbations and susceptibility to *Clostridium difficile* inferred from mouse model experimental data

In a recent study, Buffie et al. described experiments on the effect of the antibiotic clindamycin on the intestinal colonization with the spore-forming pathogen *C. difficile* [19]. The experiments were performed in a mouse model and high-throughput DNA sequencing was used to measure the relative abundance of bacterial species in cecum and ileum. The experiment consisted of three distinct populations of mice. The first population received spores of *C. difficile*, and was used to determine the susceptibility of the native microbiota to invasion by the pathogen. The second population received a single dose of clindamycin to assess the effect of the antibiotic alone. Finally, the third population received a single dose of clindamycin and, on the following day, was inoculated with *C. difficile* spores. The untreated mice challenged with *C. difficile* (population #1) did not develop infection and maintained a stable microbiota throughout the entire experiment. The single dose of antibiotic (population #2) resulted in a dramatic reduction in the microbiota biodiversity, with more than 90% of the initial species dropping below detection. The effects of this perturbation were long lasting, and biodiversity did not return to pre-treatment levels even 28 days after the clindamycin dose. Finally, mice that received *C. difficile* following the treatment with clindamycin (population #3) were colonized by the pathogen, with 40% of those mice dying due to *C. difficile* induced colitis.

The experiment was performed in three replicates: for each population three mouse colonies were uniformly treated, but separately housed. Each time point represents a mouse from each colony which was sacrificed to determine the intestinal microbiota composition. Mice from the same colony are biological replicates which justifies the interpretation of these compositions as one time line representing one co-housed mouse population [19].

We used the cecal content data to infer microbial interactions, growth rates and susceptibilities to clindamycin (see Materials and Methods). Our mechanistically-based model presupposes absolute abundances. Therefore, we converted the normalized DNA sequence abundances obtained by metagenomics by multiplying with the number of universal 16S rRNA per gram of cecal content (measured using qPCR) multiplied by the sample density, 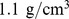 [52] (the actual density value has little importance for the inference of the interactions given the model scaling invariance, see Materials and Methods). For consistency with the previous study [19] we integrated only the ten most abundant genera including the pathogen *C. difficile*, together accounting for the vast majority (approx. 90%) of the total sequences obtained from 16S rRNA high-throughput DNA sequencing (Figure S4). The remaining lower abundance microbes were grouped into a category called “Other” (see Materials and Methods). This choice resulted in less than 30% of undetected entries in the data matrix. The choice of a higher number of independently treated genera, e.g. 15, could result in more than 50% of missing values in the data matrix (Figure S5).

**Figure 2:**
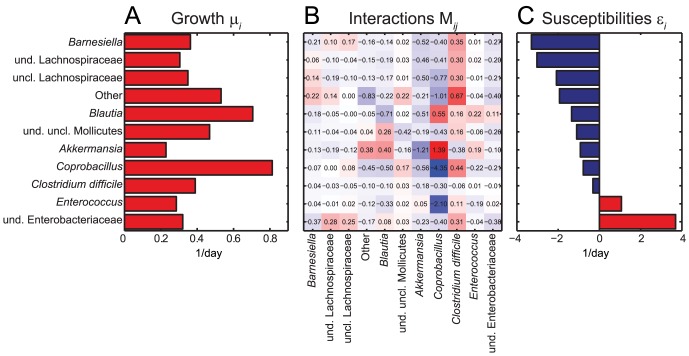
Growth and interaction rates and susceptibilities to clindamycin application from cecal mouse data. All growth rates are found to be positive (A). Interaction parameters in row *i* and column *j* represent the effect of genus *j* on *i* where red stands for activation and blue for repression (B). Blue bars in the susceptibility panel refer to an inhibiting effect of clindamycin, while red ones refer to activation (C). The optimal regularization parameters obtained in a 3-fold cross-validation are 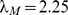, 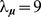, 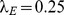.

Consistent with the underlying biological assumptions, the specific growth rates obtained from our inference method (Figure 2A) are all positive, and concordant with values measured *in vitro* using representative species of human colonic microbiota (0.55–1.78 per day [53] compared to 0.2–0.9 from Figure 2A). The diagonal elements of the obtained interaction matrix (Figure 2B) are negative. This is again consistent with the underlying biology, since it means that each of these species would eventually reach carrying capacity even in the absence of other species.

*Coprobacillus* is found to be the genus with strongest interactions by value in the ecological network. Specifically, it appears to primarily inhibit all the other microbes, including *C. difficile*, with the exception of *Akkermansia* and *Blautia* which also show inhibitory effect on *C. difficile*. The strongest inhibitory effect is on *Enterococcus* which together with the group of unclassified Mollicutes is inferred to positively interact with the pathogen *C. difficile*. This positive association is consistent with previous reports [54], [55]. Intriguingly, our method also suggests *Barnesiella* to mildly inhibit *Enterococcus*, which agrees with previous findings in mice and humans [25]. Susceptibilities to clindamycin (Figure 2C) propose that the antibiotic inhibits all of the genera, except for *Enterococcus* and the group of undefined Enterobacteriaceae. *C. difficile* itself is mildly repressed by the antibiotic.

### Stability of the intestinal microbiota

**Figure 3:**
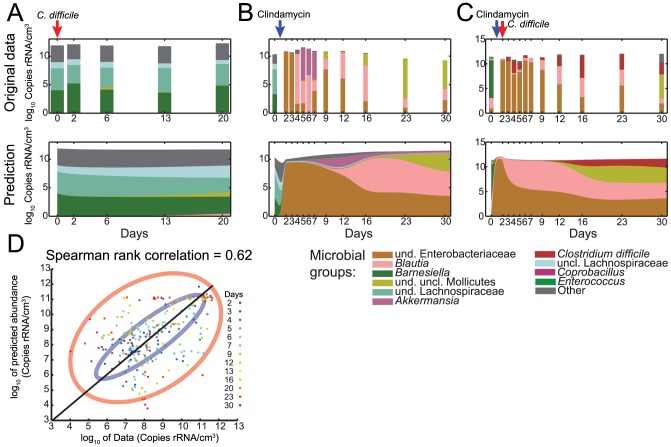
Comparison between observation and predicted microbial composition in the cecum. (A) refers to replicate 2 of population #1 (*C. difficile* inoculation at day 0), (B) to clindamycin administration at day 1 (replicate 2 of population #2) and (C) to clindamycin and *C. difficile* administration at day 1 and 2 respectively (replicate 2 of population #3). The composition bar is linearly scaled. Note, the total abundance of the intestinal microbiota does not decrease with antibiotic treatment. This may indicate the specific function of the bacteria that are present after the perturbation. (D) Rank correlation of measured with predicted data points. Colors indicate elapsed time. 75% confidence ellipses are drawn for the first (blue) and last (red) predicted time points.

Next, we investigated the implications of the inferred model parameters for microbiota dynamics. First, we tested the model's performance in predicting microbiota trajectories. To do so, we inferred the growth, interaction and susceptibility parameters on 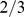 of the available data, leaving 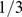 of the trajectories to test the model prediction. Subsequently, we solved eq. (1) numerically using the inferred parameters, initial compositions and the metadata of antibiotic and/or *C. difficile* inoculation (see Materials and Methods for further details). In Figure 3, we compare the observed dynamics of the second replicates with the dynamics inferred from the first and third replicate. Figure S6 shows the full comparison for all the three replicates. The simulated trajectories show a good agreement with the experimental data for all the three populations with respect to order of magnitude and qualitative behavior. There are, however, discrepancies especially in Figure 3B. Here, the experimental data shows a community take-over of *Akkermansia* and *Blautia* three days after clindamycin treatment. Our method predicts the same behavior but with several days delay (see Discussion for possible explications and model limitations). The rank correlation between data and prediction is of 62% along time (Figure 3D).

We then investigated the long-term stability of the system. We calculated the steady-state composition of the microbiota, 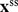, as a solution of eq. (1) for vanishing the time-derivatives in the absence of any perturbation. Consequently, there are 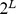 steady states where 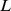 is the number of microbial groups in the system. Of these, one state corresponds to the trivial case of total extinction (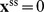), one state corresponds to the case of total coexistence (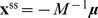, for 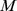 invertible), and 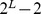 states correspond to the permutations of existence or extinction for every other species [56]. A priori, we have no knowledge about which one of these 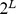 states the system will attain. This depends on the initial composition, presence and duration of the external perturbations. Therefore, we determine the steady state by simulating long-term dynamics to obtain information on species extinction and coexistence. Once this information is obtained, we can analytically evaluate the steady state of the system and its qualitative behavior by determining the spectrum of the corresponding Jacobian matrix evaluated in that state (see Materials and Methods). The principle of linearized stability states that if the real part of the largest eigenvalue of the Jacobian is negative then the composition 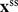 represents a stable microbiota (an asymptotically stable state). Otherwise, it is unstable [57]. For instance, the total extinction state, 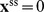, is unstable if any of the growth rates is positive, which is true for our data (Figure 2A). However, the dynamics of high-dimensional Lotka–Volterra systems allow for a large variety of different qualitative behaviors such as limit cycles, chaos or attractors [39].

**Figure 4:**
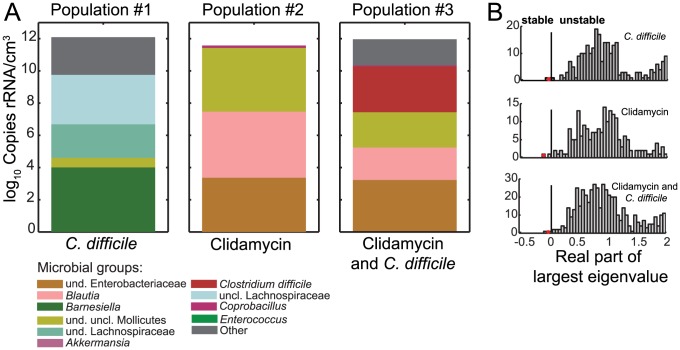
Steady state microbial composition for the cases described in Figure 3A–C. (A) predicted composition of the second replicates of the three different populations. These states are asymptotically stable as depicted in (B) where the corresponding largest eigenvalues of the Jacobian matrix evaluated at each steady state is compared (red dot) against the histogram of largest eigenvalues of all attainable and biologically meaningful steady states.

We applied this analysis to our system and identified one unique steady state for each independent replicate (Figure 4A). The replicate corresponding to untreated mice challenged with *C. difficile* (population #1) is characterized by high abundance of clindamycin-sensitive bacteria such as *Barnesiella*, undefined Lachnospiraceae and unclassified Lachnospiraceae. The steady state corresponding to clindamycin application (population #2) is characterized by a take-over by *Blautia*, unclassified Enterobacteriaceae and unclassified Mollicutes. Finally, for the case corresponding to *C. difficile* after clindamycin (population #3), the steady state predicts severe *C. difficile* colonization in addition to the genera emerging in population #2. Interestingly, these steady states agree in order of magnitude, community profiles and composition with the last experimentally measured data point of Figure 3A–C. However, in the observed trajectories the composition still changes between the last two observed data points. This could be due to the fact that the microbiota is not yet stabilized (i.e. still in transient dynamics) or due to the effect of fluctuations [15]. Although this cannot be discerned from a simple observation of the data, assuming that our model captures the actual microbiota ecology our analysis suggests that the microbiota of the perturbed microbial communities did not recover their original composition within 28 days from treatment cessation. Rather, the microbiota stays in distinct, perturbation-history dependent equilibria. The intact microbiota is, by antibiotic administration, driven towards a composition which is more susceptible to *C. difficile* colonization. By subsequent introduction of the pathogen, the community is dragged into an alternative stable composition including the otherwise repelled *C. difficile*; this may be an example of “niche opportunity” [58], [59]. Interestingly, when considering the landscape of all possible steady states of the inferred Lotka–Volterra model, unstable steady states, i.e. those referring to critical compositions which drive communities with similar compositions to a collapse or catastrophic shift [60], are significantly more often observed than stable ones. Given the inferred parameters, we find that of the 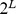 steady states which the system is able to attain from a composition of *L* initially present genera, about 98% are found to be unstable (Figure 4B). Nonetheless, our model predicts the existence of multiple stable compositions in each of the three experimental arms. Our results, therefore, may indicate the existence of alternative stable compositions of the intestinal microbiota; switches between these states are induced by perturbation with clindamycin or *C. difficile* inoculation. This concept is reminiscent of ecological stability and resilience discussed by Connell and Sousa [61].

### Subnetwork conferring protection against *C. difficile*

**Figure 5:**
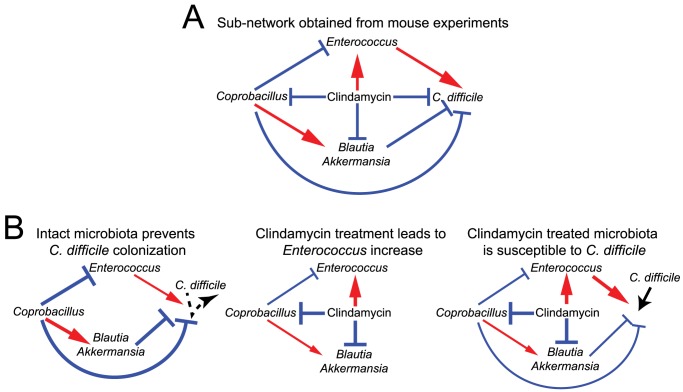
Colonization mechanism. (A) Mechanism of *C. difficile* colonization in mice. (B) Schematics of step-by-step dynamics leading to *C. difficile* establishment following clindamycin treatment.

The inspection of the model inferred from mouse experiments [19] could suggest a possible ecological mechanism for *C. difficile* colonization (Figure 5A). In the intact microbiota, our method proposes that *Coprobacillus* interacts positively with the genera of *Akkermansia* and *Blautia*. Additionally, *Coprobacillus* inhibits *Enterococcus*, which, when increasing in abundance, enhances *C. difficile* establishment. Without clindamycin, the three genera *Coprobacillus*, *Akkermansia* and *Blautia*, maintain intestinal stability and confer resistance against *C. difficile* colonization (Figure 5B). However, when clindamycin is administered, *Coprobacillus*, *Akkermansia* and *Blautia*, are inhibited while *Enterococcus* is promoted. As the three protective groups decrease in abundance, our results suggest that *Enterococcus* increases in abundance and may facilitate colonization by *C. difficile*. We discuss the validity of this mechanism in the Discussion section.

## Discussion

We presented a general method for the inference and prediction of multispecies ecological community dynamics under perturbations. Although this method was primarily developed having in mind the intestinal microbiota, the same method may be potentially applied to time-resolved data from any ecological systems, such as bioreactors [62], marine [63] or soil ecosystems [64].

Our method quantifies growth rates, community interactions and susceptibilities to external perturbations in a single inference. The modeling approach is based on the generalized Lotka–Volterra model (eq. (1)), a system of non-linear ordinary differential equations, whose governing parameters can be stably determined by a regularized regression on the discretized version of the model (eq. (2)). Microbiota metagenomics data often have a high number of microbial species which is much larger than the number of available time points. This presents a challenge to inference. We solved this problem in two steps. The first step was to group the bacterial sequences at the genus level of phylogenic classification and consider only the ten most abundant microbial genera including the pathogen *C. difficile* and merge all remainders to “Others”. The second step was to apply Tikhonov regularization, a procedure that provides a unique and stable solution and, in combination with cross-validation, reduces the risk of overfitting noisy data. Our inference method was tested using *in silico* data (Figure S1) and evaluated by its ability to recover left-out data using a cross-validation approach (Figures S2, S3).

The application of inference methods to temporal metagenomic data shows great promise. Still, the development of accurate, predictive models, for example for clinical application, will require further developments and the next few years are sure to see major improvements in this area. For example, the method used here to group microbial sequences may be expanded by adding functional information in addition to taxonomic information. Future methods will benefit from deeper sequencing of the metagenome [65] to inform new ways to define functional microbial groups. Such analyses can shed new light, for example, on the mechanisms by which the abundance of certain species seem to correlate with susceptibility to colonization by closely related pathogenic bacteria [66]. Regarding antibiotic effects, even though we are not yet able to measure the effective concentrations of the antibiotic in the intestine in a high-throughput manner, more accurate information on the pharmacokinetics *in vivo* will greatly enhance the applicability of this method to clinical settings. Likewise, experimental advancements with animal models will also be crucial. The experiments analyzed here consisted of a single dose of clindamycin of 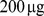 by intraperitoneal injection [19]. Comparing antibiotic perturbed mice with intact mice in this case is similar to comparing a thriving forest with one that has burnt to the ground. The same antibiotic administered in gradual dosages, or the use of other antibiotics, will surely produce distinct effects and would allow for analyzing the communities with distinct compositions. Also, engineered artificial microbiota with defined numbers of bacteria in germ-free mice could be a valuable tool to test the resilience of communities with increasing complexity. Longitudinal data collected from such experimental models can give valuable new insight into the mechanisms of protection against *C. difficile*.

Other differences between data and simulation results may stem from approximating the infinitesimal by time-discrete dynamics and the fact that the Lotka–Volterra model incorporates only pairwise, second-order interactions (eq. (1)). This could be relaxed in the future by extending the model to third or higher-order interactions once more data becomes available. Furthermore, due to the requirements of the Lotka–Volterra framework our method cannot be applied directly to read count data without additional information on the total number of bacteria per volume unit. If this information is not available it needs to be estimated which can be a source of deviations between measured and predicted results. Nevertheless and even though we cannot claim that the inferred interactions are revealing real causative relationships among microbes, we believe that our results go beyond the explanatory power of widely-used correlations and other methods used. A major advantage of this method is its foundation on a mechanistic framework. This allows for the determination of directional interactions as well as the simulation of microbial dynamics with considerable agreement with the actual data.

Based on our inference results, we also hypothesized on a mechanism of *C. difficile* colonization. However, making a substantiated statement on this mechanism would require further analysis across different host systems and under various antibiotic perturbations. Moreover, due to the limited phylogenetic resolution of the 16S rRNA sequencing, our approach would assign the effects of possibly few, interaction-mediating strains to the whole genus. Nevertheless, the analysis presented here suggests possible experiments focusing on the role of *Enterococcus*, *Coprobacillus*, *Blautia* and *Akkermansia* in mediating *C. difficile* colonization. This could be investigated, for example, in mice with engineered microbial consortia. Specifically, the microbiota of these mice could be manipulated to lack the genus of *Enterococcus* or to contain after antibiotic treatment representative strains of genera such as *Coprobacillus*, *Blautia* and *Akkermansia* which are predicted to have protective effect. Non-colonization and clearance of *C. difficile* in this system after clindamycin application would then support our hypothesized infection mechanism.

There is an urgent need to understand how the commensal intestinal microbial community resists invasion by pathogenic species. Mathematical modeling and inference can help shed new light on this problem by disentangling the contribution of each factor at play. The combination of increasingly accurate and affordable sequencing methods with solidly grounded mathematical theory can help advance our understanding of the relationship between the human host and its microbial inhabitants.

## Materials and Methods

### Model

A general approach for a deterministic model of time-dependent ecological dynamics is given by the following system of autonomous coupled first-order ordinary differential equations, in which each time course represents the time-variation in abundance, 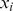, of an ecological unit 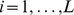 in a certain environment,
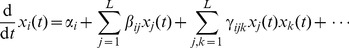
(4)with unknown parameters, 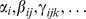 for 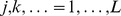. A requirement of ecological models for closed systems is that a unit that once goes extinct cannot return. Thus, for unit 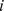 which is extinct at time 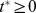, we require 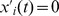 and 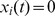 at any time 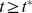 independent of any variation of the remaining 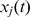, 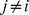. In the framework of (4), this necessitates 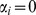, 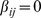 for 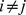 and 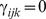 for 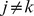 such that, if we restrict to only pairwise interactions, we obtain for each unit 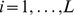,
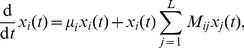
(5)where 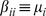 and 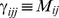 for 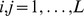. This system of equations is also known as the Lotka–Volterra model [39]. The 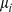 denotes the unlimited growth rate of unit 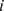 in absence of any competition. The interaction term 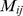 characterizes the effect of unit 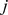 on 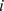. In particular, 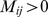 stands for activation and 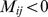 for repression. (No interaction is accordingly indicated by 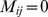). In this form, the model, which is governed by the absolute abundances of units and their physical, order-dependent interactions, also captures non-linear dynamics such as Monod-type/Michaelis–Menten kinetics in a first-order approximation. In addition to growth and interactions we introduce the effect of the application of 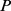 external time-dependent stimuli, 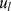, on each ecological unit such that the full model writes,
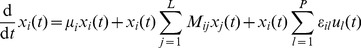
(6)where 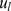 represents an external, time-variable stimulus of a perturbation 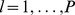 whose relative susceptibility for each unit 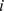 is represented by 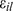.

In the framework of metagenomic data, one faces large magnitudes of total numbers of bacteria. A common approach to identify scale-dependencies of the system and to circumvent numerical problems associated with this is to use non-dimensional variables which allow to treat the model relative to changes on typical system scales [67]. For this purpose, we introduce the following representation of the dynamical variables,
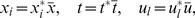
(7)where the dimensionless forms are denoted with asterisks and the barred variables denote the typical scales of the variables. For the measurements of the intestinal microbiota used in our analysis, we find typical scales for abundance and time of 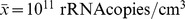 and 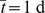. Equation (1) then reads in dimensionless form as,
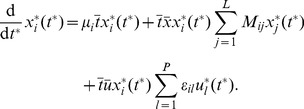
(8)We choose the scale for the perturbation signal such that it is scaled to 1, i.e. 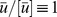. Thus, we obtain the rescaled growth rates, interaction parameters and susceptibilities as, 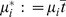, 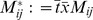 and 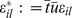 and recover the original equation (1) by dropping the asterisks. Given this choice, the (rescaled) parameters of growth and susceptibility are found to be scale-invariant of changes in the typical abundance 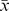, in contrast to the interaction parameter 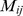.

### Parameter inference and prediction

Input variable is one longitudinal data-set in time points 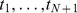 with abundances of 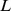 taxonomic units (in the following analysis, genera), 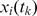, and 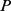 time-dependent perturbations represented by their signal 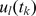. The parameters of interest are the growth, interaction and susceptibility parameters, 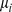, 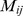 and 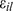.

#### Discretization and linear problem

In order to use the time-discrete data in the infinitesimal framework of the Lotka–Volterra system, we rewrite eq. (1) for 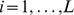,
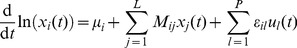
(9)using 
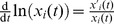
 for 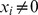. For given values 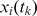 at 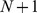 discrete time points 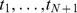, the time derivative in (9) can be approximated by the forward difference quotient,
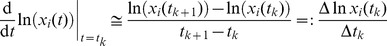
(10)for 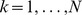 and using 
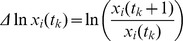
. Note, there is no limitation to equally-spaced time steps. Accordingly, the discretization of the full equation (1) is then given by,
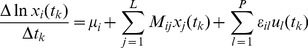
(11)for successive time points 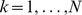. Note, the data points assigned to the last time, 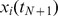 and 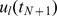, have to be removed from each trajectory. We regroup (2) in linear equation system employing the whole time-series information. For this purpose, we adopt a matrix notation for the unknown model parameters, the interaction matrix 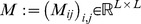, the susceptibility matrix 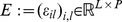 and the growth rate vector 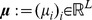. Moreover, we tabulate the time-series information on the species' abundances and perturbations in 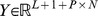 and the corresponding difference quotients in 
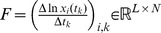
, which yields for (2),
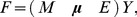
(12)where
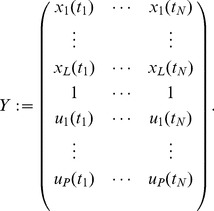
(13)Note, equation set (12) is invariant to simultaneous flips of the columns of 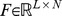 on the left and of the data matrix 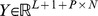 on the right hand side, i.e. the column order can be changed as long as it is done on both sides. This allows us to infer global parameters of interaction, growth and susceptibility, 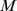, 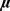 and 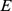 on multiple data sets by concatenating several time-series trajectories. In practice, equation (12) is analogously valid when the data matrices *F* and *Y* are filled with *S* independent trajectories each consisting of 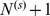 data points, 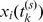, at time points 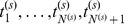 for 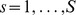 and 
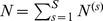
.

#### Parameter estimation and model validation

Equations of type (12) are commonly solved by regularized linear regression with a suitable regularizer. This approach in combination with a suitable model evaluation reduces the risk of overfitting by finding the optimal trade-off between model complexity and predictability on unseen data [68]. For our problem, we use a Tikhonov regularization (also known as 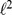-regularization or ridge regression) with its standard formulation as minimization problem (3) with positive penalty terms 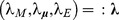. This biases the solution towards smallness of the parameters 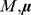 and 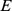 relative to the square of the 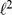-norm 
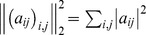
. The 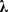-dependent solution is then given by,
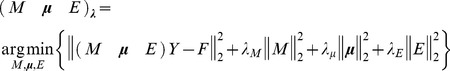
(14)

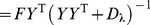
(15)with the diagonal matrix 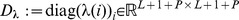 with entries 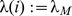 for 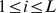, 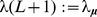 and 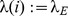 for 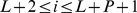. Since 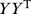 is positive-semidefinite, 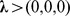 is sufficient to guarantee that 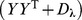 is invertible and thus (15) has a unique solution.

To this point, 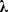 can be chosen to select the set of 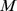, 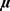 and 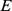 which predicts best on unseen data. A standard approach to address this is to apply 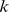-fold cross-validation in which the data is randomly partitioned into 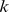 equally sized subsets: 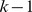 of these are used to infer the parameters 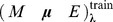 using (15) for several combinations of 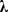. The remaining, unseen subset is used to estimate the corresponding prediction errors, 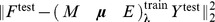. This is repeated for all 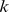 possible partitions into 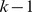 possible training sets and one test set. To reduce random fluctuations, several rounds using different random partitionings are performed. Based on the results of this procedure, we choose 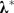 as the penalty parameter with the minimal averaged prediction error on unseen data. The final model is determined by applying 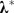 to the complete data set. It is representative of the system's parameters and has been selected for best predictive performance on unseen data. In simulations on artificial data, we find that this procedure with 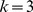 and ten runs of cross-validation recovers successfully the model parameters (which are known in the case of *in silico* data), see Figure S2.

#### Prediction of trajectories and stability of steady states/long-term behavior

The long-term behavior of trajectories in Lotka–Volterra systems is determined by the model's steady states (also referred to as equilibrium or fixed points). The Lotka–Volterra equations are non-linear and therefore allow for the existence of multiple steady states. Any trajectory in a certain environment of an asymptotically stable steady state solution tends to approach this state in time. Principally, this also applies to solutions under perturbations and allows the system to stay in or recover its original configuration. This behavior can be compared to some extent with the notion of resilience, i.e. the ability of a system to keep or recover its original state after perturbations, in the context of ecological systems.

The global 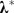 enables us to predict the dynamics of unseen systems given only the initial composition and the time-dependent signal of perturbations and/or introduced taxonomic units. For this purpose, we use the obtained parameters 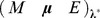 determined on the full data set except for the to be predicted trajectory (or all trajectories of a certain group). Given the obtained parameters, we subsequently solve eq. (2) numerically using the initial values of the to be predicted trajectories.

In particular, the steady states of system (1), 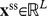, are determined by 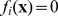 for 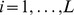 with 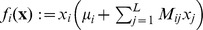. Their qualitative behavior is characterized by the spectrum of the corresponding Jacobian matrix evaluated in that state,
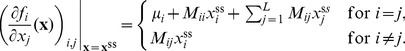
(16)The principle of linearized stability states that, if the real part of all eigenvalues of the Jacobian is negative, then 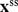 is asymptotically stable. Otherwise, it is unstable [57].

### Application to *in vivo* metagenomics data

The operational taxonomic units counts per sample and relative phylogenetic profile as presented in [19] were used as input data for our analysis. As described in the Results section, we considered the ten most abundant genera (including the pathogen *C. difficile*) and a group “Other” containing the remaining lower abundance genera. The particular grouping was used to reduce sparsity in the data matrix and to avoid spurious, presumably noise-driven contributions. The choice of using the genus level for phylogenetic resolution was dictated by the fact that 1) it is consistent with the original published paper [19] and 2) it represents the most specific phylogenetic level for which we have classification data. In our grouping, we denote a microbial genus “undefined” (abbreviated with “und.”) when the phylogenetic classification was non-ambiguous up to a certain phylogenetic level.

In contrast to Buffie et al. [19] in which the data of the three replicates are presented by their average, we use the individual nine time courses from the cecum (three from each colony) and concatenate their compositions spanning 86 time points into the data matrices 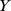 and 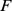. In case of non-detection of an otherwise present genus, we assign a uniformly distributed random value between zero and the detection limit of the corresponding sample. Whenever a genus is completely absent from all considered samples in a particular inference, its corresponding row in the data matrix 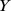 of above is set to zero. The perturbation signal for clindamycin is modeled by a unit pulse of length 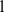 day centered on the time of antibiotic administration.

Subsequently, the inference was performed as described above with 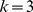, i.e. in every round of cross-validation, six of the nine time courses were used as training and the remaining three as test set. Ten rounds of cross-validation yielded the minimizing regularization parameter 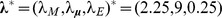. The result for 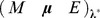 using all the data of nine time courses is presented in Figure 2.

In the next step, we predicted the behavior of known trajectories only using their initial compositions and clindamycin application and/or *C. difficile* inoculation and compared it to the measured values. We used 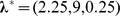 from above to infer 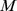, 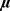 and 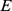 on six out of the nine trajectories, two from each population. These parameters were used to solve eq. (2) numerically for the remaining three trajectories only providing initial compositions and perturbation profiles and/or *C. difficile* inoculation. Figure 3 shows the predicted trajectories of the second replicate of each of the three populations using parameters inferred on the remaining six.

Moreover, the same parameters were used to assess the stability of the three steady states by linear stability analysis (see above). In Figure 4, we compared these to the final composition of the corresponding measured time courses.

#### Computational resources used

Inference and prediction algorithms were implemented in MATLAB R2012b (Mathworks Inc., Natick, MA). Numerical integration of the ordinary differential equation systems were performed using the native function ODE15s. Simulations were run in the cluster facility at cBio@MSKCC.

## Supporting Information

Dataset S1 Microsoft Excel file reporting data (processed taxa densities as well as antibiotic profiles), optimal regularization parameters and inferred model parameters.
(XLSX)

Figure S1 Typical *in silico* trajectory of studied species with superimposed white noise and under application of three random perturbations as used in the *in silico* validation of our method.
(EPS)

Figure S2 Comparison of stepwise prediction error vs. error on inferred parameters for variation of the regularization parameters. It can be seen that the minimum in stepwise prediction error and error in parameters approximately coincide.
(EPS)

Figure S3 Generated *in silico* input data for inference (blue symbols) and superimposed trajectories obtained by inference and re-run of the ordinary differential equation system (black lines).
(EPS)

Figure S4 Histogram of time-averaged abundances of the ten most abundant genera (including *C. difficile*) and “Other”. The grouping covers 90% of all detected individual genera.
(EPS)

Figure S5Coverage of the remainder group “Other” (blue) considering 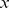 distinct most abundant genera compared to the fraction of entries below detection limit in the data matrix (red).(EPS)

Figure S6 Comparison of measured data and predicted time courses. The column number of each time line determines its replicate number (1 to 3) and the row number points to which population it belongs (#1 to #3). In particular, of the nine trajectories, A, D and G correspond to the population inoculated only with *C. difficile* spores, B, E and H to the ones only treated with clindamycin and C, F and I to the cases in which the mice were treated with clindamycin and subsequently inoculated with *C. difficile* spores.
(EPS)
